# An Analysis of Kikuchi Lines Observed with a RHEED Apparatus for a TiO_2_-Terminated SrTiO_3_ (001) Crystal

**DOI:** 10.3390/ma14227077

**Published:** 2021-11-22

**Authors:** Jakub Pawlak, Marek Przybylski, Zbigniew Mitura

**Affiliations:** 1 Faculty of Physics and Applied Computer Science, AGH University of Science and Technology, al. Mickiewicza 30, 30-059 Kraków, Poland; jakub.pawlak@agh.edu.pl (J.P.); marprzyb@agh.edu.pl (M.P.); 2 Academic Centre for Materials and Nanotechnology, AGH University of Science and Technology, al. Mickiewicza 30, 30-059 Kraków, Poland; 3 Faculty of Metals Engineering and Industrial Computer Science, AGH University of Science and Technology, al. Mickiewicza 30, 30-059 Kraków, Poland

**Keywords:** perovskities, nanostructured materials, interfaces, SrTiO_3_, RHEED, Kikuchi patterns, inelastic scattering

## Abstract

In this study, electron diffraction patterns observed under high vacuum conditions for an SrTiO_3_ surface were interpreted in detail while paying special attention to the features of inelastic effects. The surface of the SrTiO_2_ was carefully prepared to enforce its termination with single domains of TiO_2_ layers at the top. The inelastic patterns were interpreted using analytical models. Two types of Kikuchi lines are recognized in this paper: those which can be described with the Bragg law and those which appear due to surface wave resonance effects. However, we also discuss that there exists a formal connection between the two types of the Kikuchi lines observed.

## 1. Introduction

If a beam of electrons with energies of 10–50 keV hits a crystal surface, then diffraction of the electron waves occurs, and diffraction patterns on respective screens can be observed. Some bands (lines, etc.) called Kikuchi effects may appear, which cannot be explained directly with the concept of plane wave diffraction; it is necessary to assume that some electrons are first incoherently scattered into all directions and only then diffracted. Such effects were found, both in transmission and reflection geometry, soon after the discovery that electrons may behave as matter waves (in particular, for reflection experiments, respective results were discussed by Nishikawa and Kikuchi in [1]). The incoherent scattering of incident electrons may happen due to thermal vibrations of the atomic nucleus in a crystal. It became apparent that the occurrence of incoherent thermal effects (also known as inelastic, phonon scattering effects) can be in fact very helpful with respect to obtaining information about the structure of investigated samples. In particular, using electron backscatter diffraction (the technique developed in combination with scanning electron microscopy), microstructural details of the arrangement of atoms in samples can be extracted (for example, see [2,3,4]).

To obtain information about nanoscale crystalline surfaces, researchers often use reflection high-energy diffraction (RHEED). In some respects, RHEED is like electron backscatter diffraction. However, for the case of RHEED, grazing geometry is applied (i.e., the value of the angle between the incident beam and the surface is typically less than 5°, whereas for electron backscatter diffraction, this angle is several times larger). For RHEED, it is relatively rare to carry out the analysis of inelastic features caused by thermal vibrations because this type of regular effect can be observed in the grazing geometry only if the surface of a sample is very well ordered (otherwise, surface imperfections cause stronger but less regular effects). However, it seems that a deeper understanding of phonon scattering features is becoming more and more important even for RHEED because nowadays, nanostructured samples are much more refined than those fabricated 40 years ago, when the use of RHEED started to become popular (in combination with molecular beam epitaxy).

It is worth summarizing important works on Kikuchi effects appearing in RHEED patterns described in the literature. Larsen et al. [5] analyzed patterns for ordered and partially disordered GaAs(001) surfaces. Similar surfaces were investigated by Braun et al. [6]; however, they extended their studies to also include AlAs(001). Dudarev et al. demonstrated an approximate theoretical approach in which inelastic collisions were treated kinematically, but the propagation of elastic electron waves was described more precisely, i.e., dynamically [7]. In their book, Ichimiya and Cohen [8] presented fundamental information about Kikuchi features; in particular, they displayed useful analytical formulas for determining the Bragg lines and resonance envelopes. Here, it is worth mentioning that there is also some software on the web for simulating RHEED patterns, including Kikuchi lines [9]. This software was developed by Nakahara for Apple computers. This website currently seems to be inactive; however, from the materials presented there, basic concepts on how simulated RHEED patterns should look like can be learned. Furthermore, the authors of another book [10] thoroughly discussed the fundamentals of the propagation of electron waves in crystals, paying great attention to inelastic effects. Finally, in [11], Hagiwara and Shigeta analyzed RHEED patterns for Si(111) surfaces with different reconstructions, and in [12], Sun et al. presented an investigation for growing surfaces of SrTiO_3_(001). The last two papers, containing analyses of Kikuchi effects, are quite recent, so we will discuss them in detail.

Hagiwara and Shigeta interpreted the resonance lines that they observed in experimental patterns for Si(111). Their concept that interpreting whole RHEED patterns for fixed angles of the incident beam may be helpful for obtaining details of the surface reconstructions is interesting and encouraging. However, it seems that further development of numerical software for dynamic calculations is needed to achieve the quantitative level of such analyses. Namely, an explicit inclusion of lattice thermal vibrations and/or other possible sources of incoherent scattering of primary electrons at some stage of the interpretation seems to be important.

Recently, strontium titanate (with the chemical formula SrTiO_3_) has started to attract much attention as one of the materials that can potentially be applied to future electronic devices due to its interesting properties. In any case, Sun et al. [12] carried out an analysis of inelastic Bragg lines to support the validity of their description of the growth of an SrTiO_3_ thin film with the use of molecular beam epitaxy. The experiments were conducted employing time-controlled shutters, and subsequently, SrO and TiO_2_ fluxes were supplied alternately on a TiO_2_-terminated SrTiO_3_ substrate. According to the authors of [12], the film growth was realized in the layer-by-layer mode, in which single monolayers of SrO and TiO_2_ were formed alternately at the crystal top. The periods of the RHEED oscillations observed were sometimes in direct agreement with the description proposed, but sometimes the periods were two times longer than one might expect from a simple phenomenological approach. However, according to the explanation given by Sun et al. [12], the occurrence of double periods is caused by the difference in the mean inner potential for TiO_2_- and SrO-terminated surfaces. To support their description, the authors precisely analyzed the variations in the shapes of the Kikuchi lines. Actually, such an explanation of results observed is acceptable; however, the problem still requires further investigation. This is because another explanation is also possible; namely, if the growth was realized by the simultaneous formation of two oxide monolayers, then periods of two types might also be also recognized: the basic ones and periods two times shorter due to the effect of frequency doubling (the occurrence of such an effect has been reported for a number of materials; see [13,14,15]). In any case, conclusions drawn only from the observation of RHEED oscillations cannot be unambiguous. In a very recent paper, Orvis et al. [16] demonstrated that Auger electron spectroscopy can be properly adjusted to check the composition of the growing surface termination. Their results also suggest that the alternate formation of single SrO and TiO_2_ monolayers is indeed possible. However, further investigation might aid a better understanding of the growth modes of complex oxides and also possibly help in developing useful parametrized models (in practice, growth modes are never perfect, but this can be taken into account in theoretical work; for the concept of parametrized descriptions, for example, see [17]). It needs to be emphasized that the paper of Sun et al. [12] seems to be very important for the nanotechnology of perovskite thin films, and the concept of analyzing Kikuchi patterns to learn additional information about the layers grown is valuable.

The primary goal of our research work was to learn what kind of Kikuchi effects appear for well-prepared perovskite surfaces. We carried out our investigations keeping in mind the question of whether a thorough observation of Kikuchi lines can be of some help in controlling the preparation of substrates that are applied to fabricate nanostructures. More specifically, we were interested in answering the question of whether both Bragg and resonance lines can be recognized in respective experimental patterns. This question seemed to be important in the context of recently published papers [11,12], where only single types of lines were discussed. In our work, we investigated a TiO_2_-terminated SrTiO_3_ crystal, which is an example of a well-prepared perovskite substrate.

## 2. Materials and Methodology

### 2.1. Details of Experimental Work

The RHEED measurements were conducted in a chamber that is typically used for pulsed laser deposition (PLD). The PLD method is currently quite commonly used by researchers dealing with the growth of high-quality thin films (for a review, see [18,19]). This method can be applied to prepare different perovskite films [20,21]. For the preparation of structures with complex stoichiometry, PLD can indeed be competitive with respect to molecular beam epitaxy, which is often used in nanotechnology (for example, see [22]). If PLD systems are equipped with a RHEED apparatus, then in situ examination of surface samples is possible [23,24].

The PLD system used in the current work, with base pressure of about 10^−8^ Torr, was built and accessorized by Neocera, Inc. (Beltsville, MD, USA). The high-pressure RHEED apparatus (STAIB Instruments GmbH, Langenbach, Germany) installed in the system is dedicated to monitoring the changes at the sample surface during the material deposition; subsequently, the geometry of the incident beam is fixed. However, samples can be precisely rotated around the axis perpendicular to the surface. Diffraction patterns can be observed with the help of a charge-coupled device camera (k-Space Associates, Inc., Dexter, MI, USA).

In this work, we focused on analyzing RHEED patterns that can be observed for a well-prepared surface. We investigated a TiO_2_-terminated SrTiO_3_ sample that was prepared as prescribed by Connell et al. [25]. According to their procedure, the sample should be annealed at 1000 °C in air for at least 1 h and cleaned in deionized water. The confirmation that atomically smooth surfaces can indeed be prepared with the help of such a method was shown in the paper of Pawlak et al. [26]. Furthermore, after placing the sample inside the PLD system, a pressure of about 10^−8^ Torr was obtained. The RHEED experiments were conducted at room temperature. The energy of the primary beam electrons was set to 20 keV.

It should be noted that SrTiO_3_ substrates can be further used for layered nanostructures. In some situations, it is possible to observe RHEED oscillations. However, in the current paper, we focused on discussing the results obtained without the deposition of materials, in order to avoid the appearance of structural imperfections at the surface. For TiO_2_-terminated SrTiO_3_ samples, fine details can be recognized in RHEED patterns, and thus, a detailed theoretical analysis is possible.

### 2.2. Theoretical Approach Applied

**Figure 1:**
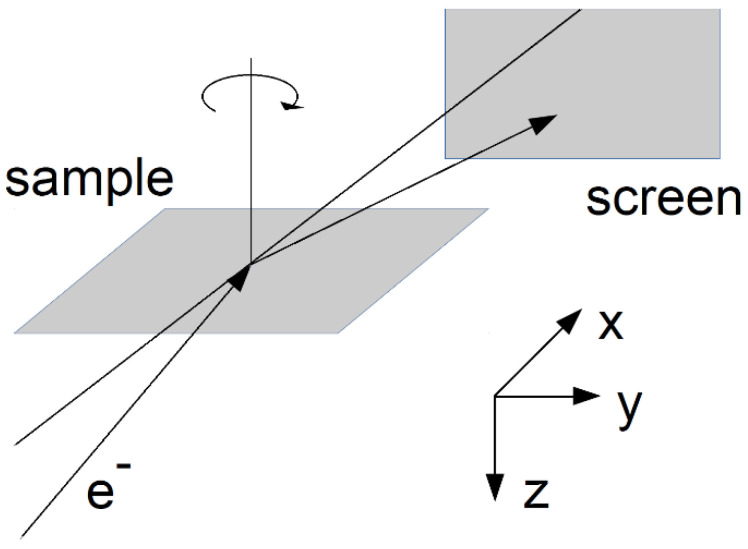
Geometry of the measurements and the system of coordinates applied in theoretical considerations.

Interpreting whole RHEED diffraction patterns is not especially simple because, after the crystal is hit by the electrons of an incident beam, multiple scatterings of the electron partial waves occur. Furthermore, the scattering events may be coherent or incoherent (for a thorough discussion, see [10]). In practice, for specific experiments, only selected phenomena can be considered in a theoretical analysis. It seems that for whole RHEED patterns, the most advanced but practically manageable approach was demonstrated by Korte and Meyer-Ehmsen [27]. They treated coherent processes dynamically (i.e., multiple scatterings of elastic waves were included in their approach), but incoherent events were treated kinematically (i.e., perturbatively). The dynamic treatment of the diffraction of elastic waves requires time-demanding computations. In our work, we decided to use another approach. We were interested in reproducing basic geometric features of the patterns, so we employed simplified analytical formulas. To interpret the Kikuchi lines, we followed the prescription given in the book of Ichimiya and Cohen [8]. The Bragg reflection lines were generated with the help of the Bragg law written in quadratic form. The resonance lines were determined using a phenomenological equation developed from the dynamic theory of diffractions for electron waves propagating nearly parallel to the surface. However, we introduced our own modification in the description of the resonance lines in relation to the treatment of Ichimiya and Cohen [8]. The aim of the modification was to make it possible to carry out a joint analysis of Bragg reflection and resonance effects. Additionally, to simulate the whole RHEED patterns, the distributions of the spots that appear at the screen also needed to be determined. We employed formulas from dynamic diffraction theory, which are results of the use of the two-dimensional (2D) Bloch wave approach. In fact, it is convenient to discuss the details of the modelling patterns starting with the explanation of the formulas for the spots. Later on in the paper, it assumed that the surface of the sample is parallel to the xy-plane (see Figure 1).

#### 2.2.1. Set of Bragg Spots

We have assumed that the appearance of a set of spots at the screen is caused by diffraction electrons scattered elastically. In fact, electrons scattered inelastically may cause similar effects if the direction of the wave propagation remains the same after an inelastic event and the change of the electron energy is of order 1%. In general, such effects may happen due to the electron–plasmon interaction; however, there is no need to consider them separately in the analysis if only the positions of the spots need to be determined.

Precise descriptions of the propagation of electron waves inside a crystal can be achieved if the Schrödinger equation is properly used in the theoretical treatment. Looking for such descriptions constitutes the subject matter of the dynamic diffraction theory for electrons. Let us assume that the surface of a crystal is defined by $z=z_{T}$ and the crystal is in a space determined by $z<z_{T}$. Furthermore, the incident beam is assumed to be a plane wave, described by the wave vector $K_{i}$. If the crystal is periodic in the planes parallel to the surface, then the electron wave function Ψ(r) can be expressed in the following form [8,10,28]:

(1) $$\Psi \left(r\right)=\underset{g}{\sum }\phi_{g}\left(z\right)\exp\left[i\;(K_{i∥}+g)\cdot \;r_{∥}\right].\;$$

In Equation (1), $K_{i∥}$ is the parallel component of $K_{i}$, and similarly, $r_{∥}$ is a parallel component of r. Due to the 2D periodicity of the crystal, the 2D reciprocal surface lattice can be defined. Respectively, in Equation (1), g denotes a vector of this lattice. It should be mentioned that Equation (1) can be treated as a starting point of the 2D Bloch wave approach developed to compute intensities of spots observed at the screen (for more details, see [8,10,29,30]). Here, we are interested only in the determination of a set of wave vectors of the beams propagating towards the screen. For $z>z_{T}$, the part of Ψ(r) resulting from diffraction can be expressed as a sum of partial waves with the form $R_{g}\;\exp\left[\;i\;(K_{i∥}+g)\cdot \;r_{∥\;}+i\;K_{gz}z\right]$, where $R_{g}$ is the amplitude of the wave. However, such terms may describe both propagating waves (if $K_{gz}\;$is real) or evanescent waves (if $K_{gz}\;$is imaginary). Only propagating waves cause the appearance of spots at the screen; therefore, we have not considered further evanescent waves.

The procedure to determine the set of wave vectors $K_{g}$ allowing one to compute the positions of the spots at the screen is as follows. Initially, for a selected vector g, we calculate x and y components of $K_{g}$ and some auxiliary value *h*.


(2) $$\{\begin{array}{c} K_{gx}=\;K_{ix}\;+\;g_{x},\\ K_{gy}=\;K_{iy}\;+\;g_{y},\\ h={\left|K_{i}\right|}^{2}-{K_{gx}}^{2}-{K_{gy}}^{2}. \end{array}$$

If *h*<0, we simply reject the selected vector g from considerations. If h>0, we can write

(3) $$K_{gz}=\sqrt{h},$$

finally having all the required components of $K_{g}$. Additionally, we need to specify all the components of $\;K_{i}$. Assuming that this vector is always fixed in the xz-plane, its components can be expressed as follows:

(4) $$\{\begin{array}{c} K_{ix}=\;|K_{i}|\sin\theta ,\\ K_{iy}=0,\\ K_{iz}=\;-|K_{i}|\cos\theta , \end{array}$$

where θ means the glancing angle.

To derive formulas to find the distribution of spots, we used some concepts from the 2D Bloch wave approach. This means that, in some sense, we employed the framework of dynamic diffraction theory. In principle, it should be possible to only use kinematic arguments and employ the Ewald sphere construction [31,32]. Therefore, we can say that elastically scattered electrons cause the appearance of “Bragg spots” at the screen. The kinematic and dynamic theories indeed give identical results if spot positions are considered (predictions of the theories differ if spot intensities are analyzed). However, to explain the appearance of resonance lines, dynamic theory findings still need to be recalled (see Section 2.2.3).

It is worth noting that the thickness of our sample was equal to about 0.5 mm. However, even for such thick samples, it is necessary to use the concept of reciprocal space rods (rather than points) to describe the part of the diffraction pattern formed by elastically scattered electrons. This part of the pattern is caused by the interference of coherent, partial electron waves coming from very few atomic layers located at the surface. This is because, in RHEED, we consider the electron waves (with the coherence limited by inelastic events) which move nearly parallel to the surface (due to the application of grazing geometry). A more detailed discussion can be found in the book by Ichimiya and Cohen [8].

Using Formulas (2)–(4), it is possible to predict RHEED patterns resulting from one-stage scattering of electrons by a well-ordered surface, i.e., if inelastic events are ignored. In Section 2.2.2 and Section 2.2.3, we discuss how to theoretically predict two-stage scattering effects.

#### 2.2.2. Bragg Reflection Lines

It is assumed that the primary beam of electrons hits the crystal surface; this beam can be described with the help of the wave vector $K_{i}$. Many electrons are scattered incoherently into all directions. After the inelastic collisions, these electrons are diffracted by the periodic crystal potential. We are interested in finding the conditions for the intensity maxima for waves reaching the screen, defined by the wave vector $K_{f}$. We assume that $|K_{f}{|}^{2}={\left|K_{t}\right|}^{2}={\left|K_{i}\right|}^{2}$, where $K_{t}$ is one of the wave vectors of the electrons scattered incoherently due to thermal vibrations (i.e., we ignore small changes of the electron energies). We apply the following equation to determine those maxima at the screen that can be assigned to the vector G of the three-dimensional (3D) reciprocal lattice:

(5) $$2K_{fx}\;G_{x}+2K_{fy}\;G_{y}+2\;{\left(K_{fz}{}^{2}-\tilde{v}\right)}^{1/2}G_{z}={\left|G\right|}^{2}.\;\;\;\;\;$$

In Equation (5), $K_{fx}$, $K_{fy}$, $\;K_{fz}$, $G_{x}$, $G_{y}$ and $G_{z}$ are the respective components of the vectors $K_{f}$ and G, and $\tilde{v}$ is the reduced mean potential of the crystal.

Some discussion of how to use Equation (5) to plot Kikuchi lines is given in [8]. Here, we briefly show a derivation of Formula (5). It is necessary to start from the Laue equation: $K_{f}-K_{t}=G$. Then, we can write $|K_{f}-G{|}^{2}={\left|K_{t}\right|}^{2}$, followed by $2K_{f}\;G={\left|G\right|}^{2}$. The latter equation constitutes some form of the Bragg law. It was employed for determining Kikuchi lines for the case of RHEED by Larsen et al. [5]. However, a very similar equation was mentioned earlier in a book by Kittel [33] in the course of the presentation of X-ray diffraction.

For the case of RHEED, the effect of the refraction also needs to be included in the analysis. This is realized by replacing the wave vector component $K_{fz}$ by ${\left(K_{fz}{}^{2}-\tilde{v}\right)}^{1/2}$. The values of $\tilde{v}$ can be determined using the following relation (derived from the Schrödinger equation):

(6) $$\tilde{v}=\left(1+\frac{|q_{e}|U}{m_{0}c^{2}}\right)\frac{2m_{0}}{\hbar^{2}}\tilde{V}.$$

In Equation (6), $\tilde{V}$ is the mean inner potential. Further, the term ($1+(|q_{e}|U)/(m_{0}c^{2}))$ describes the relativistic correction ($|q_{e}|\;$is the absolute value of the electron charge and U is the accelerating voltage of the electron gun). In general, the values of $\tilde{V}$ can be found experimentally or theoretically. We used the second method, employing parametrized Gaussian functions for electron scattering factors for ions [34] (actually, for ions, extra non-Gaussian terms also exist, but they cancel each other out in neutral crystals [35]). Subsequently, $\tilde{V}$ was determined with the help of the following formula:

(7) $$\tilde{V}=-\frac{\hbar^{2}}{2m_{0}}\;\frac{4\pi }{c_{latt}^{3}}\left(3\underset{i}{\sum }a_{i}^{O}+\underset{i}{\sum }a_{i}^{\mathrm{Ti}}+\underset{i}{\sum }a_{i}^{\mathrm{Sr}}\right),\;$$

where $a_{i}^{O}$, $a_{i}^{\mathrm{Ti}}$ and $a_{i}^{\mathrm{Sr}}$ denote the values of the respective Gaussian parameters for O^2−^, Ti^4+^ and Sr^2+^ ions [34]. Accordingly, we found that $\tilde{V}=-15.08$ eV. It is worth noting that Equation (7) can be obtained based on the definition of electron scattering factors, using their relations with the electrostatic potentials of atoms and ions (for details, see [8,10,29]).

#### 2.2.3. Resonance Lines

Surface resonance scattering is a dynamic phenomenon; i.e., its full analysis requires solving the Schrödinger equation. Bragg reflections (discussed in Section 2.2.2) have a simpler interpretation—to obtain the basic formulas, only the constructive interference of waves needs to be considered. In fact, Bragg reflection lines were already recognized in the 1920s [1]. The situation was different for resonance lines. There was a long debate in the literature on special effects which might be expected if an electron beam formed due to diffraction moved nearly parallel to the surface (see [36] and references therein). However, it seems that the situation became much clearer when the paper of Ichimiya et al. [37] was published. The authors demonstrated experimental resonance lines and formulated the conditions for their appearance. Namely, sometimes electrons can be channeled inside a crystal because of internal reflection. Ichimiya et al. [37] carried out research using the technique called convergence beam RHEED, but their results can also be generalized for the case of diffuse scattering observed with the standard RHEED apparatus when primary beam electrons move in one direction (for a detailed discussion, see the book of Ichimiya and Cohen [8]). Therefore, in our current work, we used concepts from the aforementioned paper. However, we also introduced some modifications allowing us to discuss a formal connection between Bragg reflection and resonance lines.

We assumed that each resonance line is associated with some vector g of a 2D surface reciprocal lattice. The following formulas were used to determine the shapes of the lines:

(8) $$\left\{ \begin{array}{c} 2K_{fx}\;g_{x}+2K_{fy}\;g_{y}+K_{fz\;}{}^{2}-\alpha \;\tilde{v}\;={\left|g\right|}^{2}\\ \mathrm{and}\\ \alpha \approx 1. \end{array}\right.$$

To show the derivation of these formulas, we initially recall (as in Section 2.2.1) that due to the diffraction of waves by the periodic potential in the planes parallel to the surface, many coupled beams appear above the surface. If we assume that the beam of electrons moving in the direction defined by $K_{f}$ represents the reference beam, then we can consider a beam with the wave vector $K_{-g}$. The following relations are satisfied: $K_{-g∥}=K_{f∥}-g$ and $K_{-gz}{}^{2}=|K_{f}{|}^{2}-{\left|K_{f∥}-g\right|}^{2}$ (both $K_{-g∥}$ and $K_{-gz}$ are related to $K_{-g}$; specifically, $K_{-g∥}$ is the vector component parallel to the surface and $K_{-gz}$ is the *z* component). Now, we need to analyze the condition $K_{-gz}{}^{2}=0$, which describes the change of the form of the electron wave. For $K_{-gz}{}^{2}>0$, outside the crystal, a propagating wave appears in the formal solution of the diffraction problem. For $K_{-gz}{}^{2}<0$, the appearance of an evanescent wave can be observed. However, inside the crystal, due to the refraction, for the appearance of an evanescent wave, fulfilling the stronger condition of $K_{-gz}{}^{2}-\tilde{v}<0$ needs to be considered. Furthermore, according to Ichimiya et al. [37], if the conditions $K_{-gz}{}^{2}<0$ and $K_{-gz}{}^{2}-\tilde{v}>0$ are satisfied, the beam determined by $K_{-g}$ has the propagating wave form inside the crystal, but due to the internal reflection effect, the electrons cannot leave the crystal. Consequently, an increase in the intensity of the basic beam (with the wave vector $K_{f}$) may be expected, and due to this, a Kikuchi envelope may appear at the screen. We slightly modified this approach. First, we formulated the conditions for the envelope as the relation $K_{-gz}{}^{2}-\alpha \;\tilde{v}=0$, where the parameter α may take values between 0 and 1. Accordingly, we can write $|K_{f}{|}^{2}-{\left|K_{f∥}-g\right|}^{2}-\alpha \;\tilde{v}=$0. After a simple manipulation, we obtain $\;K_{fz}{}^{2}+2K_{f∥}\cdot g-{\left|g\right|}^{2}-\alpha \;\tilde{v}=0$ and then Equation (8). Second, we considered the results of Marten and Meyer-Ehmsen [38] and of Dudarev and Whelan [39]. In the papers of these authors, the intensity peaks appearing due to resonance scattering are rather narrow. Furthermore, in both papers, theoretical considerations referred to the formation of bound states in the crystal. Subsequently, we assumed that resonance lines can be described with specific values of α, but values greater than 1 are also possible (the scattering potential inside the crystal is not uniform; absolute values of the potential are much larger near atomic cores than between cores). We also need to acknowledge that sometimes two (or even more) close lines may appear in experimental patterns, and such lines should be described by different, specific values of α. However, it seems reasonable to assume that, initially, α≈1.

It should be noted that in the derivation given above, we used $K_{f}$ and −g rather than $K_{i}$ and +g. The use of the latter pair would be in accordance with the book of Ichimiya and Cohen [8]. However, we were interested in establishing some connection between Bragg reflection lines and resonance lines. Therefore, we adopted the same approach as in Section 2.2.2.

#### 2.2.4. Additional Remarks

It is perhaps also useful to briefly discuss what kind of diffraction patterns can be observed if, e.g., two-dimensional islands exist at the surface. Then, a series of vertical streaks appear at the screen. This happens because the diffraction spots (caused by elastic scattering) significantly change their shapes in the direction perpendicular to the surface. One can employ the Ewald sphere construction to explain this effect.

It is worth mentioning that the methodology developed in Section 2.2.1, Section 2.2.2 and Section 2.2.3 may potentially be used, e.g., for determining the details of surface reconstructions. Analyses of this type are based on the interpretation of rocking curves, i.e., plots of spot intensities recorded as functions of the glancing angle of the incident beam. For advanced analyses, the rocking curves are measured for principal azimuths, where many Kikuchi lines exist (for example, see [40]). Since each line represents a certain diffraction condition, the overlapping of many diffraction conditions occurs for such azimuths. In our opinion, a set of computed Kikuchi lines might be helpful for selecting additional azimuths for carrying out measurements of those rocking curves for which only specific conditions are fulfilled.

Finally, it should be emphasized that, in principle, our methodology can be used for samples with relatively flat surfaces. If samples are rough, then the elastic electron waves are scattered in all directions. Moreover, the respective patterns formed at the screen are strongly dependent on the specific arrangement of atoms at the surface. In such situations, regular Kikuchi lines appearing due to the thermal vibrations cannot be easily identified in the diffraction patterns.

## 3. Results and Discussion

### 3.1. Interpretation of Experimental Patterns

**Figure 2:**
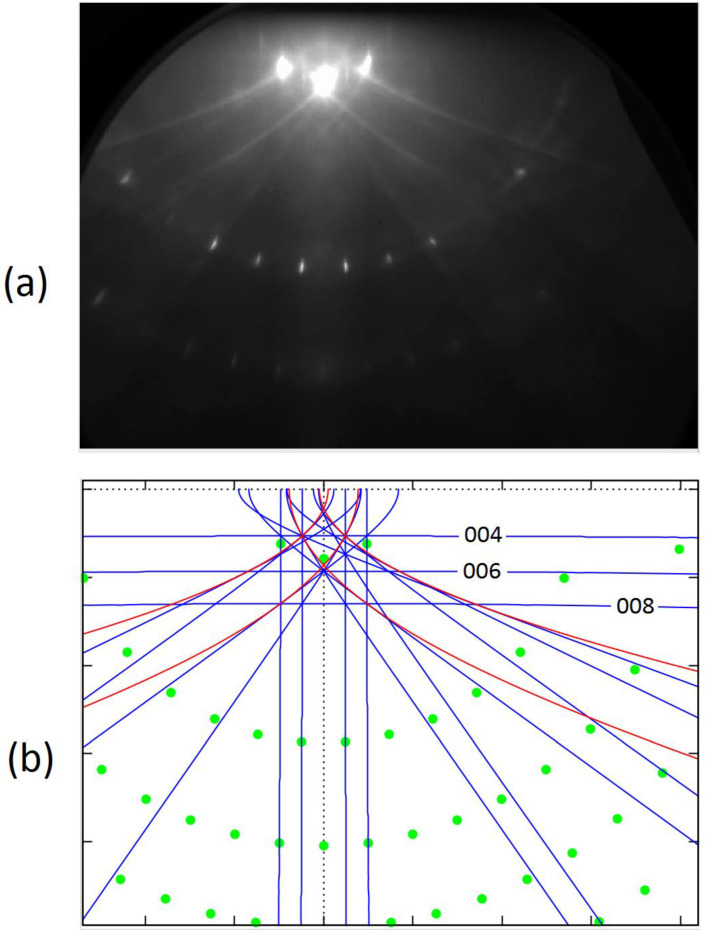
(**a**) The experimental RHEED patterns for the incident beam azimuth [110]. (**b**) The corresponding pattern determined computationally for the glancing angle equal to 2.9°. The green spots represent the diffraction of elastic electrons, while the blue and red lines represent electrons scattered inelastically (the Bragg reflection effects are shown in blue and the resonance scattering effects are shown in red).

**Figure 3:**
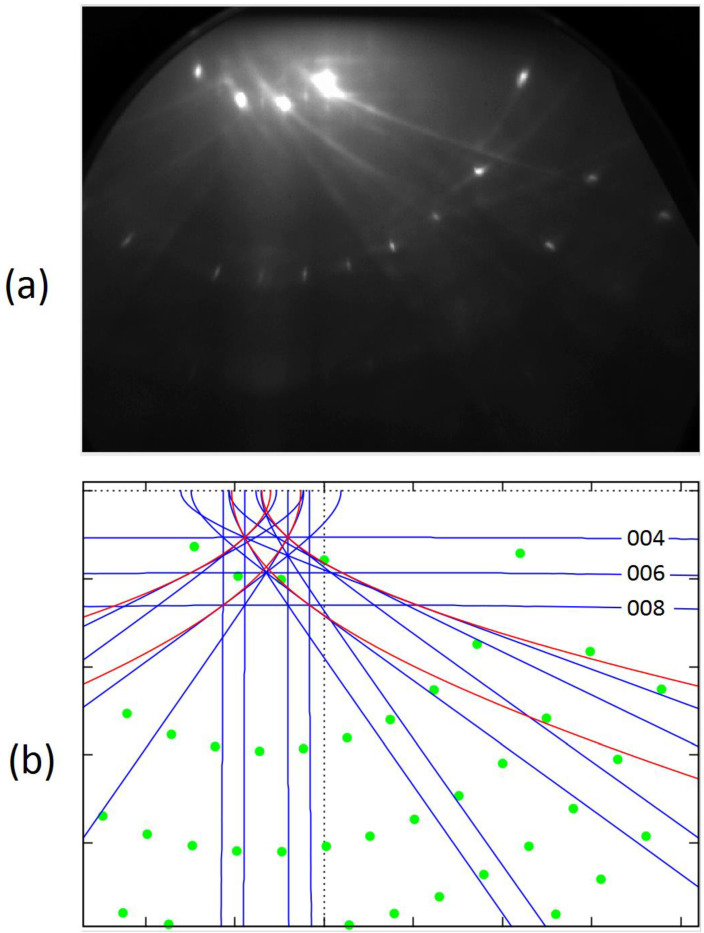
Similar to Figure 2, but an off-symmetry experimental pattern is shown in (**a**) and the theoretically determined pattern is displayed for the incident beam azimuth [110] −2.4° in (**b**).

We carried out a detailed analysis of the collected data (for the description of the experiments conducted, see Section 2.1). Below, we summarize our main findings (see Figure 2 and Figure 3).

(1) The experimental patterns contained both spots and lines. We determined the distributions of the spots theoretically, assuming that they appear due to simple diffraction of a certain part the primary beam electrons (i.e., it can be said that spots are the result of the one-stage process). The shapes of the lines were determined assuming that some electrons were first incoherently scattered into all directions and only then diffracted (i.e., in our treatment, the lines appear due to the two-stage process). In this work, we were able to achieve a very good agreement between experiment and theory. In this context, it should be noted that the polar and azimuthal angles defining the direction of the incident beam were determined computationally in a fitting procedure. The values of these angles can be measured only with limited precision, i.e., with errors of some 0.2°. In any case, the final results are very promising. It can be concluded that fine geometric details of RHEED patterns for well-prepared samples can be reproduced with the help of analytical formulas.

(2) For the SrTiO_3_ crystal with the well-prepared TiO_2_ surface, we could recognize in the experimental patterns both Bragg reflection lines and resonance lines (caused by inelastic events as described in Section 2.2.2 and Section 2.2.3). In fact, the Bragg reflection lines, far away from the shadow edge, can be observed as straight lines; the resonance scattering is responsible for the appearance of parabolic lines. The difference can be clearly recognized while rotating the sample. In any case, we were able to detect the effects of both types.

(3) Here, it seems particularly important to consider the question of what happens to RHEED patterns if the sample is rotated around the axis perpendicular to the surface (i.e., if the azimuth of the incident electron beam is varied with respect to the surface lattice). We observed experimentally (and this was confirmed in the theoretical analysis) that the distribution of the spots at the screen changes dramatically. Surprisingly, the distribution of the lines remained relatively stable—most lines moved slowly to the left- or right-hand side, but their shapes remained similar. However, when the azimuth of the incident beam was taken 1–7° off the symmetry directions of the surface, some lines were easier to recognize. Namely, the horizontal lines due to the Bragg reflections from the planes parallel to the surface appeared in the experimental patterns (compare Figure 2a and Figure 3a). Furthermore, oblique lines could be observed in a much wider angular range. It seems that precise observations of Kikuchi features may be potentially helpful in controlling the preparation of perovskite substrates and fixing their orientation.

### 3.2. Formal Connection between Bragg Reflection and Resonance Lines

Additionally, the question of how to theoretically group Kikuchi lines into some families may be considered. There is no clear answer to this question. For example, it seems quite natural to group the lines corresponding to subsets of parallel atomic planes. However, in this paper, we propose another approach. We show that the lines can be grouped into families associated with reciprocal space rods perpendicular to the surface. Both Bragg reflection and resonance lines can be included in such a grouping. This requires some additional explanation. Surface resonances can be directly assigned to rods as discussed in Section 2.2.3. However, Bragg reflections are generally determined via the Laue equation referring to 3D reciprocal lattices. In general, different sets of primitive vectors may be needed to determine the 2D surface lattice and the 3D crystal lattice for the same material. However, for SrTiO_3_, with the cubic perovskite structure, the most natural choice is to use the same vectors in the xy-plane. Subsequently, if we write the vectors G and g (these vectors were used in the discussion on Kikuchi lines in Section 2.2.2 and Section 2.2.3) as $G=\left(G_{x},G_{y},G_{z}\right)\;$and $g=\left(g_{x},g_{y},g_{z}\right)$, then we can put $G_{x}=g_{x}$ and $G_{y}=g_{y}$. Accordingly, in our case, we can easily associate a number of the G vectors with one g vector. Now, we can check the relation between the Bragg reflection line defined by some vector G and the resonance line defined by g. We need to rewrite Equations (5) and (8). However, it is now also useful to ignore the effects due to the refraction. This is because such effects are not very important in the region far away from the shadow edge of the screen, which makes our analysis become easier. After some mathematical manipulation, we can write:

(9) $$K_{fx}G_{x}+K_{fy}G_{y}=\left(G_{x}^{2}+G_{y}^{2}+G_{z}^{2}-2K_{fz}G_{z}\right)/2,$$

and

(10) $$K_{fx}G_{x}+K_{fy}G_{y}=\left(G_{x}^{2}+G_{y}^{2}-K_{fz}^{2}\right)/2.$$

We can recognize that the left-hand sides are now identical, so we can determine common points for both lines by analyzing the right-hand sides. Accordingly, we obtain the following condition:

(11) $$G_{z}^{2}-2K_{fz}G_{z}=-K_{fz}^{2}\;,\;$$

One can determine that the lines have a common point if

(12) $$K_{fz}=G_{z}.$$

**Figure 4:**
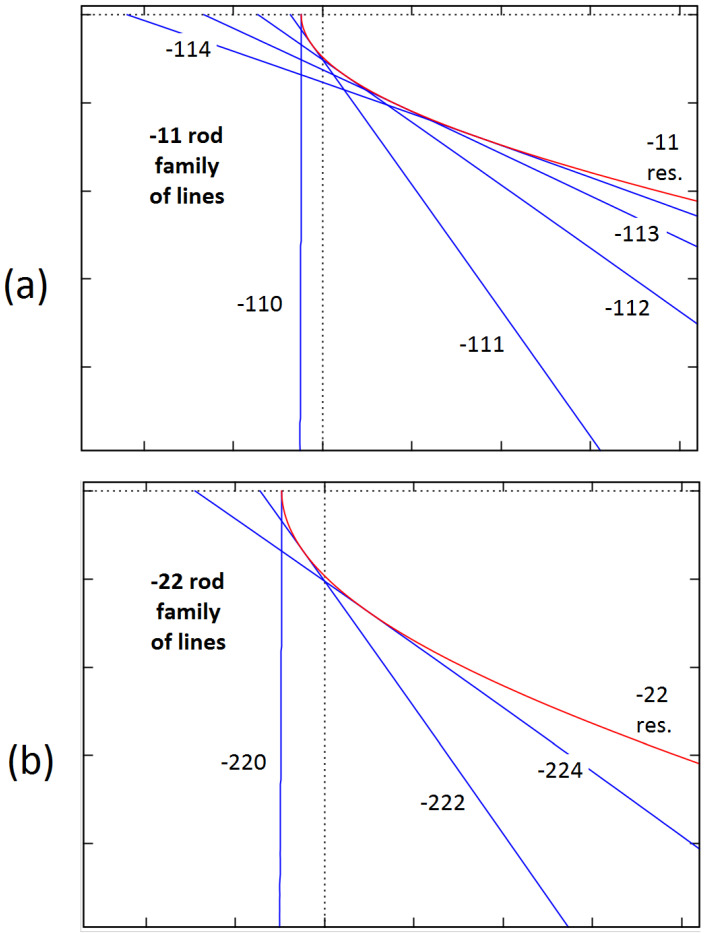
A set of Bragg reflection lines and resonance lines that can theoretically be assigned to a selected reciprocal space rod if the mean potential is ignored. Part (**a**) is for the −11 rod, while the results for the −22 rod are shown in part (**b**). The conditions of the pattern formation are assumed to be identical to those in Figure 2.

In summary, we found that the Bragg reflection line associated with the vector G has a common point with the resonance line g if the x and y components of both vectors are identical. Moreover, observing the plots of lines, it can be seen that these lines are actually contiguous (see Figure 4).

## 4. Conclusions

We found that for a carefully prepared SrTiO_3_ surface, regular Kikuchi lines of two different kinds appear in experimental diffraction patterns. This allows us to suppose that the observation of Kikuchi effects may be generally useful in controlling the preparation of perovskitie samples that can be further used as substrates in nanotechnology. It is worth emphasizing that carrying out observations of the inelastic effects is much easier if samples can be azimuthally rotated and the diffraction patterns, both for the symmetry and off-symmetry azimuths, are recorded.

In our opinion, the observations of Kikuchi lines may be particularly helpful in the case of the use of PLD systems, for which achieving ultra-high vacuum conditions is not always possible. Actually, there are many systems of this type, because the application of such conditions is costly and the operation time demanding. Nevertheless, the preparation of clean, flat surfaces under very low pressures is well understood and can be controlled, for example, by low-energy electron diffraction measurements of electron spot intensities. This is because in ultra-high vacuum chambers, the time of the self-formation of a monolayer of adatoms at the surface is of the order of a few hours [41]. The situation is less clear if samples are prepared only under high-vacuum conditions. It is known that complex oxide substrates with relatively flat surfaces can still be obtained. However, it is necessary to develop fast methods of structural characterization of such substrates. In our opinion, observations of Kikuchi effects may be helpful in this respect.

## Data Availability

Data is contained within the article.
